# Large Scale Mapping of Fractures and Groundwater Pathways in Crystalline Hardrock By AEM

**DOI:** 10.1038/s41598-018-36153-1

**Published:** 2019-01-23

**Authors:** Subash Chandra, Esben Auken, Pradip K. Maurya, Shakeel Ahmed, Saurabh K. Verma

**Affiliations:** 1 Aquifer Mapping Group, CSIR-National Geophysical Research Institute (CSIR-NGRI), Hyderabad, 500007 India; 2 HydroGeophysics Group, Institute for Geoscience, Aarhus University, Aarhus, Denmark; 3 Present Address: Jamia Millia Islamia, New Delhi, India

## Abstract

In hardrocks that cover about 20% of the Earth’s surface, it is difficult to locate steady sources for groundwater due to inadequate understanding of the fracture networks. A comprehensive knowledge of fracture distribution at the regional scale is necessary to delineate sustainable aquifers and manage them efficiently. The resistivity maps derived from the airborne electromagnetic (AEM) survey over the Ankasandra watershed in Karnataka, India, reveal sharp and deep zones of low formation resistivity, which indicate groundwater-bearing zones. It is found that some of these zones are hydrogeologically connected through fracture networks resulting in augmented yield. AEM results in combination with an in-depth understanding of the geological structures successfully map these groundwater-saturated fracture networks (or hydrogeological lineaments) that we term as ‘Hydrolins’. As groundwater occurrence is generally associated with lineaments, we analyzed the drilling and geophysical logs from 21 wells within a 380 sq.km area to study the relationships of various lineaments with ‘Hydrolins’, particularly in respect of their groundwater potential. AEM results, though calibrated and correlated with a limited number of well data, revealed a threshold groundwater horizon (TGWH), found to be at 80 m depth for Ankasandra watershed, beyond which a strong correlation exists between the depth of a well and its yield. While the TGWH may differ for different watersheds, the approach presented here can be readily adopted to map sustainable groundwater sources in hardrocks worldwide.

## Introduction

Groundwater is a prime source of fresh water globally accounting for roughly one-third of the total withdrawals of freshwater^1,2^. Large aquifers with negligible recharge have been mined for the past several decades^3^, resulting in a water crisis^4–6^, and estimates show that 21 out of 38 major aquifers in the world are facing groundwater depletion at alarming rates^7^. The crisis is severe in India, where water security is widely recognized as one of the major challenges to the nation’s economic and social development^3,8,9^. Increasing demand for water and the growing dependence on groundwater have resulted in dwindling of the groundwater resources and drying up of aquifer systems^10,11^. In this context, targeting groundwater in the Precambrian shields that mostly comprise the granitoid hardrocks is even more challenging as the groundwater occurs within narrow and often isolated fracture zones. Large parts of the tropical countries, most of them developing, are occupied by the Precambrian shields and are facing acute water shortage problems. Efficient location of narrow fracture zones in hardrocks is crucial to successful groundwater targeting. In hardrock terrains groundwater occurs within a limited depth column of secondary porosity caused by weathering and fracturing processes^12^. As weathered zones have a relatively higher porosity than underlying fractured bedrock, they have traditionally been the most important horizon for groundwater resource development. However, in India, over-exploitation has caused the weathered zones to dry up in many cases and groundwater has to be withdrawn from the underlying fissured and fractured bedrock that has limited porosity. The fractures provide all or most of the porosity, and effective permeability, which commonly decreases with depth^12^. Thus, the successful mapping and management of groundwater resources in hard rock aquifers entails delineating fracture networks and recharge zones, and in optimal siting of sustainable wells in the fractured aquifer system.

Groundwater pathways in hard rocks are usually controlled by fractures, joints, geological contacts, shear zones, faults, vugs, and other discontinuities. Their multifaceted interrelationship controls overall aquifer dynamics^13^. The fractures in hard rock are generated by several processes such as cooling stresses in the magma, tectonic activity^14^, lithostatic decompression^15,16^, and the weathering process^17,18^. In highly foliated gneisses or schists, the orientation of the fractures can also be controlled by the rock structure^2,19^. The bedrock thus usually consists of dense horizontal as well as crosscutting vertical to sub-vertical fractures with depth-decreasing density. Despite the fact that bedrock fractures are thin, associated weathering widens their effective aperture and enhances the effective porosity. Saturated fractures and weathered rocks are therefore good electrically conductive targets for AEM methods.

The classical approach to fracture mapping is to use interpolation between limited numbers of sparsely located borehole observations, which often yields an oversimplified assessment of the complex aquifer dynamics. In this context, fast and inexpensive regional geophysical surveys like aeromagnetic (AM) and/or AEM are effective for characterizing the subsurface from micro-to-mega scale. Initially the AM methods were used as a regional mapping tool and to aid hydrocarbon and mineral exploration. Subsequently their combination with the AEM measurements provided an efficient tool to explore for conductive sulphide minerals. Refinements in the instrumentation, processing and modeling techniques further established the singular or joint applicability of the AM and AEM methods for groundwater exploration.

Applications of the AM methods for groundwater exploration are based on the premise that fractures, lineaments and geological contacts are the favourable zones for the occurrence of groundwater. State of the art developments in the AM methodology facilitate effective mapping of these features and successful targeting of groundwater resources^20–25^.

Historical reviews and latest developments of the AEM methods, application of helicopter borne EM (HEM) methods to hydrological exploration for both the frequency domain (FEM) and time domain (TEM) methods as well as successful applications of multi-frequency HEM for groundwater exploration can be found in various publications^26–33^. More recently, these methods have been applied for a variety of problems like: saltwater intrusions^34,35^, freshwater pockets in tsunami-affected regions, pollution threat to aquifers^36^, mapping faults associated with aquifers^34^, hydrogeophysical problems^37^. Applications of time domain AEM methods covering various aspects of groundwater studies are also reported^9,38–44^.

The above mentioned applications elucidate the applicability of the AEM and AM methods in studying various aspects of groundwater exploration. Many of them deal with the delineation of structures, lineaments and fractures primarily with the objective of locating associated groundwater resources. However, a systematic study of the depth-wise distribution of fracture networks facilitating hydrological connectivity, the main thrust of the present work, has not been reported so far. Many of the recent AM studies clearly demonstrate how detailed mapping of magnetic lineaments can provide clues to target potential groundwater resources. Yet all lineaments and fractures may not necessarily be associated with groundwater. Also, there may be hydrogeological settings with fractured bedrock even in the absence of a lineament^45^ as shown in the present study.

A variety of electrical resistivity methods has been employed to locate water-bearing fracture zones in hardrocks^46–51^. As fractures are hard to detect, attempts have also been made to map them employing a combination of geophysical methods^45,52,53^. These approaches have been successful to a certain extent in mapping fractures on a local scale. However, they are cumbersome and expensive for mapping bedrock fracture networks at regional scale. The distribution of fractures and their groundwater potential in hardrocks is random and warrant a high density of measurements.

In the present study, we have used AEM data in combination with magnetic and borehole data to map the network of fractures in a crystalline hard rock area of 380 sq. km in Ankasandra micro-Watershed, Karnataka. The AEM results provide the key to understanding the large-scale fracture network and help in locating sustainable groundwater sources in the area.

## Study Site

**Figure 1:**
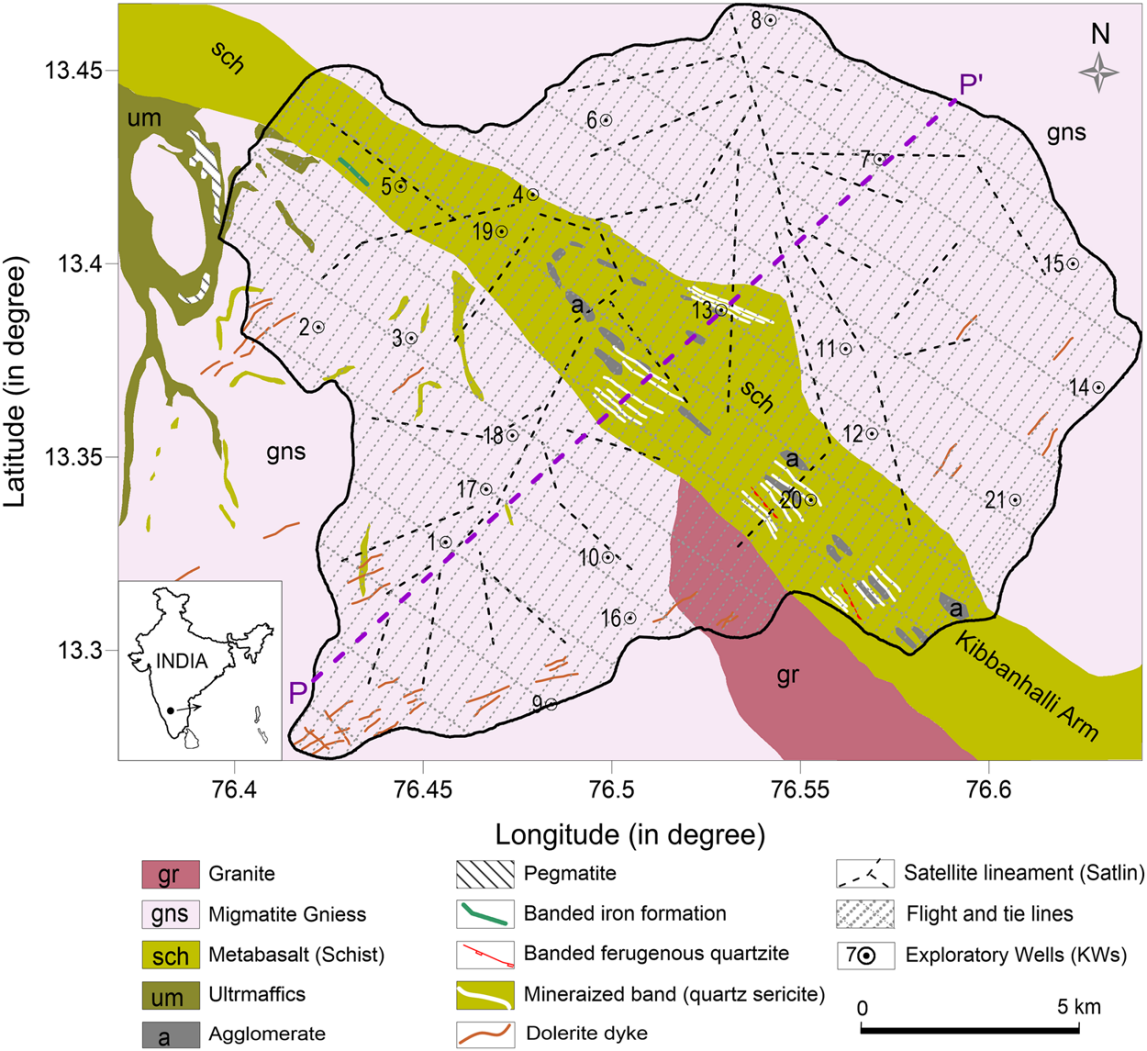
Geology of the Ankasandra Watershed (After^74^) with major lineaments, drilled wells, AEM flight lines and profile line (PP′).

The Ankasandra watershed mainly comprises Archaean Peninsular gneiss (GNS) belonging to Dharwar Super Group^54^ (Fig. 1). A NW-SE oriented meta ultramafic schist belt of the Sargur Group bisects the area into two nearly equal parts. The figure also features agglomerate, quartz sericite (QS), banded ferruginous quartzite (BFQ), banded iron formation (BIF) within the schist belt and dolerite dykes in the watershed mapped during the present study. The schist belts in the Dharwar craton are known to have sulfide mineralization (auriferous, at places) due to hydrothermal solutions and associated alterations in the QS, BIF and BFQ^55,56^, and movement and migration of mineral-rich fluids through the shear zones^57^. The mineralization continues up to a few kilometers with varying concentration along the strike of bands and shear zones within the schist belt^58^. The micro-watershed also has a few quartz veins in the central part and younger basic intrusives in the southern part. The gently undulating topography ranges from 720 m to 940 m above sea level with an average elevation of around 850 m. The area receives erratic rainfall with mean annual precipitation around 680 mm, of which around 50% occurs during the monsoon season. Groundwater is the only available water source and has been over-exploited by as much as 158% of the annual recharge^59,60^.

## Methods

We employed an AEM survey using a dual-moment SkyTEM304 system^61^, which facilitates measurement of the transient electromagnetic (TEM) response of the subsurface from very early times (~10 microsecond) to late times (up to 10 milliseconds), providing shallow to very deep subsurface information simultaneously. The system also recorded the magnetic field variations. Since the area is complex with significant geological and hydrogeological variations, closely spaced flight lines with 145 m separation were chosen and a total of 2,840 line km were flown to cover the watershed. The average flight speed was 60 km/hour while the average height of the sensor was at 35 m from the ground surface. The resulting data sampling after processing is approximately 30 m along the profiles.

The magnetic data was processed as follows: (1) stacking to a sampling of 10 Hz; (2) shifting the measurement positions of the moving magnetometer mounted on the frame to the frame center point; (3) corrections for diurnal variations, heading effect, and international geomagnetic reference field (IGRF); (4) data leveling; (5) reduction to pole and calculation of vertical and horizontal first derivatives. Finally, lineaments were delineated from maps using total magnetic intensity (TMI) and derivative data.

The AEM data was processed using the following major steps^62^: (1) importing of the stacked raw data for both the moments (low and high) and applying moving average filters, correction for transmitter altitude and tilt, processing system geometry, etc.; (2) removal of noisy data accounting for visible geomorphological and geological setup and power line distribution; (3) laterally-constrained inversion (LCI)^63,64^ along the flight lines and filtering of the noisy data with high data residual. The AEM data, after laterally constrained inversion (LCI), was inverted employing the scheme of spatially-constrained inversion (SCI)^50,54,65^ using a smooth model description with 30 layers and finally, the depth of investigation (DOI) was calculated^66^. Since the present study is significantly based on the concept of DOI, its behavior in different geoelectrical settings is briefly described below.

### Depth of Investigation

Following the diffusion principle, the skin-depth (*δ*) for a TEM signal due to a uniform primary field^67^ is given by

1 $$\delta =\sqrt{\frac{2\rho t}{{\mu }_{0}}}$$

where *ρ* is the resistivity of the earth, *μ*_0_ is the permeability of free space and *t* is the delay time. The above equation implies that the penetration is very deep in highly resistive earth, while it is shallow in conductive formations. This equation provides a qualitative estimate of the depth of penetration as it is based on uniform field approximation and does not take into account the actual measurement system, layering of the subsurface, sensitivity of the equipment and ambient electromagnetic noise, etc. For this reason, a measure of the DOI was developed based on the sensitivity matrix of the final output model of the inversion^66^. The sensitivity matrix takes in to account factors such as the layered conductivity structure of the subsurface, the system transfer function, the flight height of the sensor and the noise estimates of the data. In addition, the DOI is purely data driven as model regularization is removed before the calculation. DOI is highly dependent on the signal and noise level, but for a constant noise level it can, in general terms, be assessed as (1) limited in highly resistive terrains where the signal is low and decays very fast to “drown” in noise at very early times, (2) good in moderate resistivities, and (3) limited in very conductive terrains where the signal is high but the skin-depth is very moderate.

### Major Lineaments

Lineaments are important as the groundwater occurrence is closely associated with them. In this study the following four types of lineaments are considered: (1) Satellite lineaments, (2) Magnetic lineaments, (3) Hydrological lineaments and (4) Mineralogical lineaments. The satellite lineaments were obtained from the satellite imageries and are termed as ‘Satlins’ in this paper. The magnetic lineaments were derived from the total magnetic intensity (TMI) map of the region that revealed a number of linear dipolar anomalies. These are termed as ‘Maglins’ here. The groundwater saturated networks that provide hydrogeological connectivity to DOI patches are already described as ‘Hydrolins’. The AEM results also showed a number of linear conducting features coinciding with the sulfide mineralization in the schist belt. These are termed as mineralogical lineaments or ‘Minlins’. Relationships among various lineaments and their potential in delineating sustainable groundwater sources are studied in detail.

The AEM results were interpreted in conjunction with the lithology and the geophysical logs from 21 wells in the watershed. Fourteen wells had water quality data based on geochemical studies. Also a few (5) of them had isotope and age data. These were analyzed to study the connectivity of water from select wells with the shallow surface water. Finally, correlation between yield of wells with depth was studied that elucidated the most significant aspect of the study dealing with location of sustainable wells in hardrock terrains.

## Results

**Figure 2:**
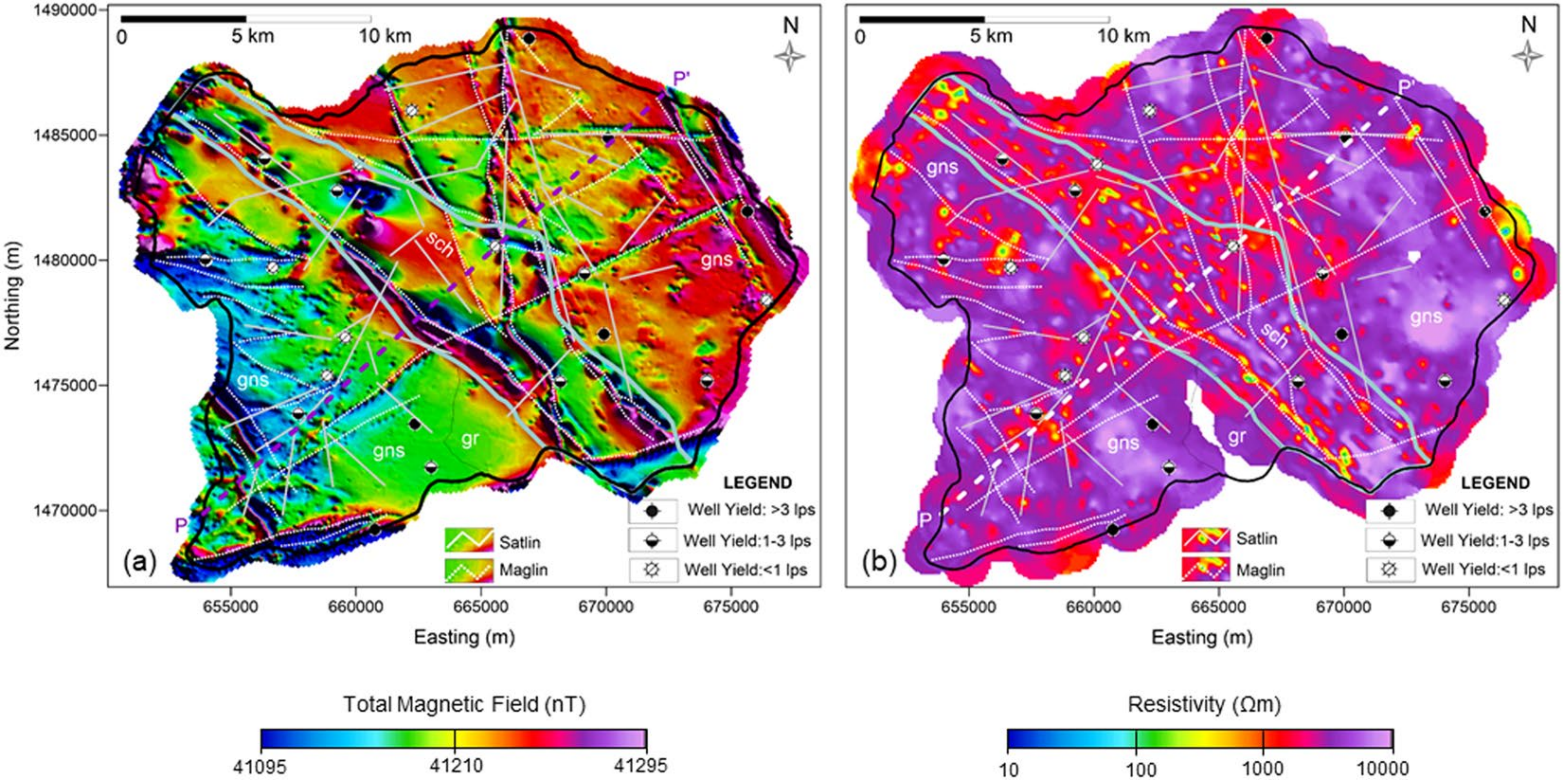
(**a**) Total magnetic field intensity map (nT). The grey line demarcates contact between gneiss and schist (schist in the center). The dashed lines in white show the magnetic lineaments. (**b**) Mean resistivity map at 100 to 110 m depth below ground level. The 21 wells are categorized in three yielding categories i.e. high (>3 lps), moderate (1–3 lps) and low (<1 lps).

The TMI values range from 41095 nT to 41295 nT, showing good correspondence with major geological features such as dikes, schist belts, quartz veins, etc. The contact of gneiss and schist is clearly seen as dipolar anomalies in Fig. 2a. The data also reveals a number of dipolar linear features striking in NW-SE, NE-SW, EW and NS directions, which are shown by the white dotted lines denoted as ‘Maglins’. They are usually associated with structure, tectonics, and/or abrupt variations in lithology and are significant indicators of groundwater storage^68^.

Only a few Maglins were known prior to the survey. The lineaments striking in N-S, NNW-SSE directions reveal a discontinuity with a noticeable shift at the point of intersection with other Maglins (for instance at points marked as A, B and C) and hence they are interpreted as concealed faults that are potential groundwater flow zones. This is validated by high-yielding wells such as KW-7, 8 and 15. High-yielding well KW9 is located at the contact of the dolerite dikes and gneisses and KW12 is close to the schist-gneiss contact. However, other wells located over Maglin and geological contact viz., KW-2, 4, 11 and 13 yield moderate or low groundwater discharge. The well KW11, though located over a major Maglin attributed as fault, yields only moderately. Thus, knowledge of Maglin and geological contacts are not always adequate to locate a high-yielding well. Possibly, they do not necessarily have the same potential all along their path. Similarly, out of 5 wells located over or close to ‘Satlins’ (KW4, 5, 7, 10 and 18), 2 wells yield high, 1 moderate and 2 low groundwater discharge (Table 1). Thus characterization with other parameters like electrical resistivity is necessary to improve the location of wells with high yield.

**Table 1:** Site specification of boreholes in terms of geology, associated lineament, drilled depths, water yield, number of fractures encountered, and corresponding AEM DOI.

UTM X	UTM Y	WELL NO	Geology & Lineaments	Drill Depth (m) bgl	Cumulative Yield (LPS)	DOI m, bgl	Fracture (F) # encountered at depth (m) within an interval of ± 0.5 m						
							F1	F2	F3	F4	F5	F6	F7
657705	1473813	KW1	Gneisses & Satlin	200	2.91	40	100.8	187.0					
654004	1480001	KW2	Gneisses & Maglin	200	1.79	ND	60.9	66.5	155.5				
656673	1479698	KW3	Gneisses	200	0.08	ND	192.5						
660131	1483823	KW4	Gneiss-schist contact	200	0.59	100	135.4	179.3					
656340	1483975	KW5	Schists & Satlin	200	1.22	100	191.5						
662437	1485946	KW6	Gneisses	200	0.08	40	68.5	88.5	110.9				
670111	1484854	KW7	Gneisses & Maglin	200	4.36	100	150.5	159.5	186.0				
666926	1488858	KW8	Gneisses & Maglin	200	4.36	110	102.5						
660768	1469202	KW9	Gneisses-Dolerite dyke, & Maglin	200	4.36	200	171.1	185.7					
662376	1473419	KW10	Gneisses & Satlin	200	3.84	90	189.5	198.5					
669171	1479394	KW11	Gneisses & Maglin, Satlin	189	1.79	50	82.5						
669929	1476968	KW12	Gneisses	200	5.54	250	55.0	77.5	96.5	111.5	128.5	143.5	161.5
665561	1480274	KW13	Schists & Maglin, Satlin	200	0.22	150	77.5						
676451	1478363	KW14	Gneisses	181	0.54	40	77.5	161.5					
675651	1481947	KW15	Gneisses & Maglin	200	4.27	170	95	120	165				
663005	1471726	KW16	Gneisses	200	1.20	70	—	—	—				
658842	1475389	KW17	Gneisses	200	0.00	20	—	—	—				
659585	1476929	KW18	Gneisses & Satlin	46.5	0.71	20	18	20	26	28			
659250	1482767	KW19	Schist	90	2.94	40	11	28					
668172	1475139	KW20	Schist	51	2.50	30	13	16	41				
674040	1475176	KW21	Gneisses	40.5	1.00	30	31	37	30				

Formation resistivity is found varying widely from 10 Ωm to 10,000 Ωm showing good contrast between various hydrogeological units. The range of resistivity derived from AEM and well-lithologs for various litho-units are listed as: (i) 15–1,500 Ωm for the top soil and unconsolidated weathered material, (ii) 10–120 Ωm for the weathered layer, (iii) 100–3000 Ωm for the fractured layer, and (iv) ≥3,000 Ωm for the compact bedrock. The upper weathered zone varies in thickness from 5 m to 30 m below the ground surface. At greater depths, resistivities are as high as 10,000 Ωm between 25 m to 250 m, indicating properties of hard and compact bedrock. This is corroborated by the borehole data from the study area as well as by published research articles on similar geological settings^40^.

The resistivity of subsurface geologic materials depends largely on weathering, fracturing, moisture content, mineralogy and compactness of the rock matrix, as well as overburden material. The analysis of AEM results brought out two zones with distinct resistivity behavior: (1) Low resistivity patches with deep DOI values in the gneisses over the water-filled fracture zones and (2) Linearly-oriented mineralized bands in the schist belt showing low resistivity and deep DOI values (Minlins).

The high yielding wells, KW7, 8 and 9, are located on Maglins and show resistivities of about 400, 700 and 5,000 Ωm respectively at 100 m depth (Fig. 2b). The background resistivity in the compact rock in these locations is more than ~10,000 Ωm. The well KW11 located on a Maglin and with a resistivity of ~700 Ωm has only moderate yield (Fig. 2b). Although wells KW10 and KW12 do not fall on any Maglin and are not in significant low resistivity zones, they still have high water yield. This suggests that there are some additional factors–most likely, a network of fractures–that contribute significantly to the hardrock hydrogeology.

## Discussions

**Figure 3:**
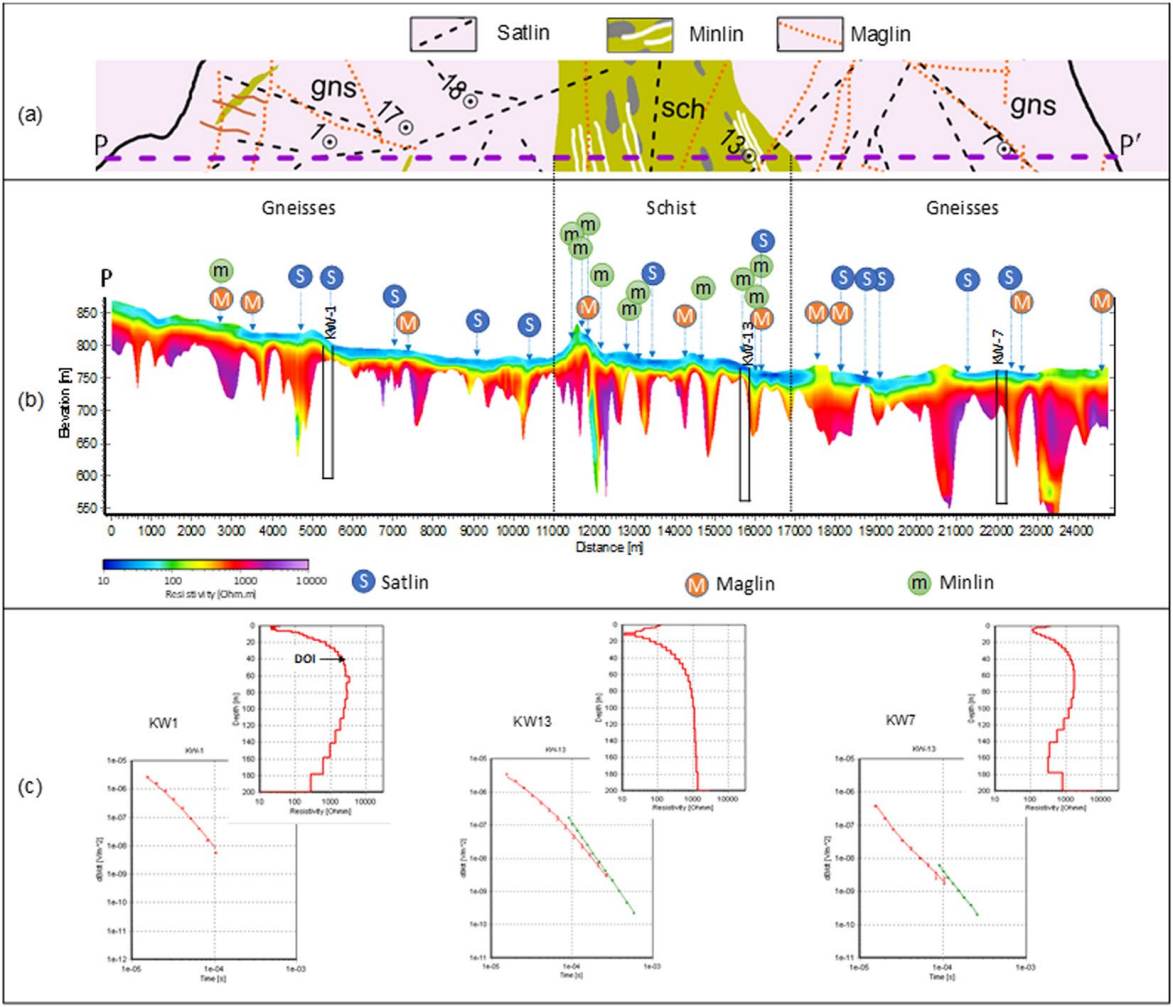
(**a**) Geology adjacent to profile PP’ marked in Fig. 1; (**b**) AEM resistivity section along the profile (PP′) passing through wells KW1 (south west) and KW13 (middle) and KW7 (north east). The resistivity section is masked below the depth of investigation (DOI). Geological contacts, lineaments and mineralized zones are also projected on the map. (**c**) AEM response (db/dt – time plot) at wells KW1, KW13 and KW7 respectively from left. The match between field and inverted data with error bars and corresponding smooth depth resistivity models are also shown. The red and green curves represent low and high moment response.

Figure 3 shows the geology along the profile line (PP′) and the corresponding AEM resistivity section crosscutting the area and passing through wells KW1, 13 and 7. The resistivity models were masked below the DOI. In general, the weathered zone is found to be varying between 20 to 40 m in thickness. The underlying bedrock shows several sharp vertical resistivity zones (steep conical troughs in isolated patches) extending to depths up to 300 m. These patches are interpreted as electrically conductive water-saturated fracture zones in gneiss. The location of high-yielding well KW7 coincides with one of these patches (formation resistivity ~370 Ωm at 100 m depth). The KW13 located in the schist zone has low yield.

Poorly yielding KW1 is located at a low DOI patch in gneiss. Figure 3(c) shows a few AEM sounding responses in the form of db/dt vs. time and depth vs. resistivity curves obtained by smooth inversion along with DOI values corresponding to wells KW1, 13 and 7. Since KW1 is located in shallow bedrock with no connectivity to deeper fractures, only low moment data was available after processing, and DOI is limited to 40 m. In contrast, both low and high moments data are available for wells KW13 and 7 due to the deep conductive subsurface setup, as they fall in the schist and deep water saturated fracture zones respectively.

As mentioned earlier, a deep DOI indicates a high degree of model sensitivity extending to greater depths. We interpret deep DOI in gneiss areas as deep-seated water-saturated fracture zones. Analysis of DOI patterns at different depth levels reveals that some of the water saturated fracture zones are hydrogeologically connected (Hydrolins) and thus have enhanced potential for groundwater.

**Figure 4:**
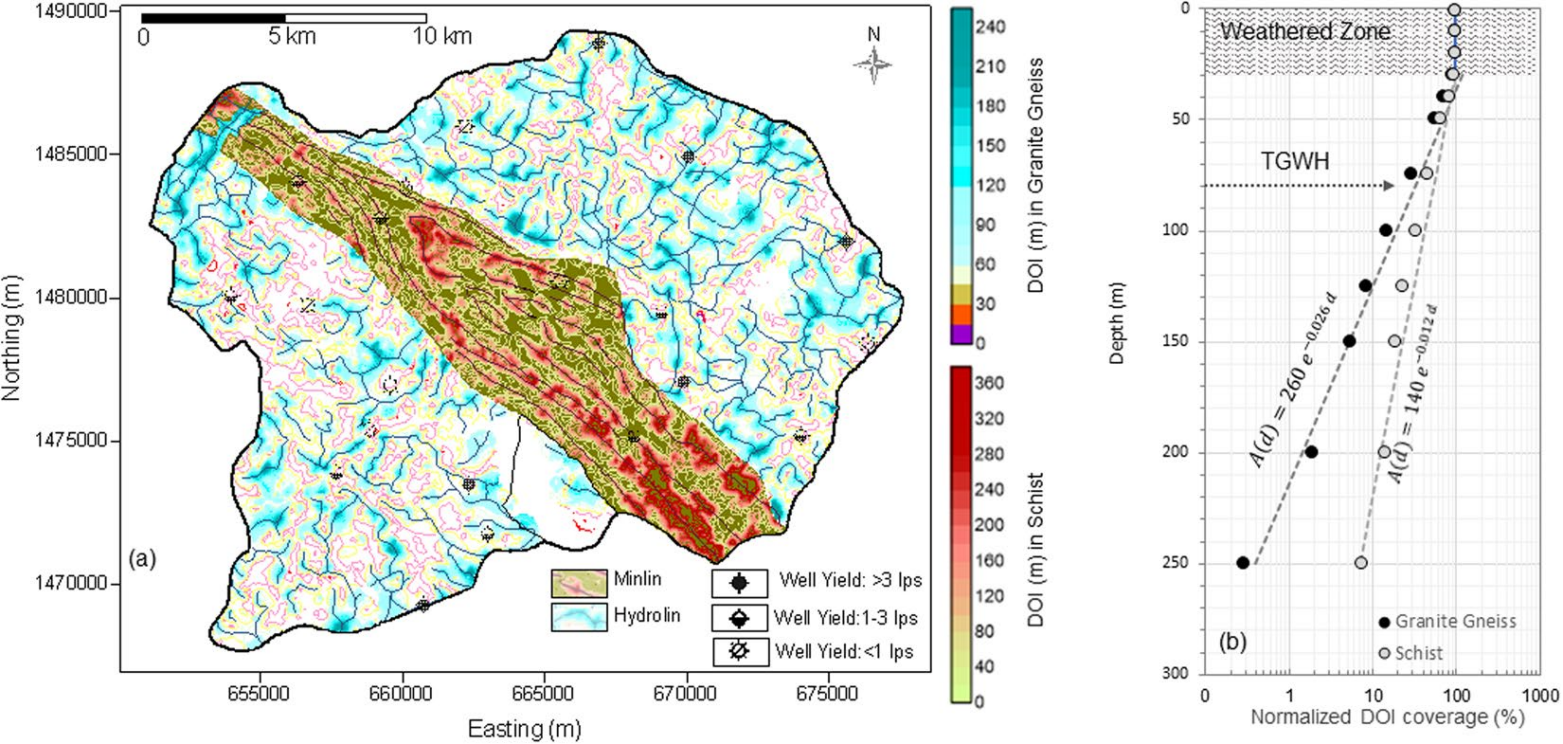
(**a**) Hydrolin and Minlin constructed using DOI contours at 100 m depth, (**b**) Normalized percentage coverage of DOI patches with depth and model curve fitting the DOI coverage data.

The DOI contours, representing the groundwater filled fracture zones and Hydrolins in gneiss and mineralization bands in schist, are found shrinking with depth in both the formations. The areas covered by these contours for both the formations are digitized at every 10 m from ground level to 50 m depth, at every 25 m down to 150 m and at every 50 m up to 250 m. Depth dependence of these areas, expressed as normalized percentage of total area of granite gneiss and schist respectively, are shown in Fig. 4b. The top weathered and altered zones for both the lithologies are about 25 to 30 m thick and show very little (<10%) areal reduction at the base. For deeper levels the general decrease can be described as shown  in eq. (2) below:

2 $$A(d)=k.{e}^{-\propto .d}$$

where *A(d)* is the normalized DOI area at depth *d*, while *k* and ∝ are constants specific to the survey area and lithology that can be derived by fitting an exponential decay curve to the observed data. For Ankasandra watershed these values for gneiss are:

*k* = *260 and* ∝ = − 0.026 while for schist the values are: *k* = *140 and* ∝ = − 0.012. Figure 4b clearly shows that the areal reduction of DOI patches in gneiss is faster as compared to that in schist. The reduction in schist can be ascribed to thinning and/or discontinuation of mineralized bands. However, from the view point of groundwater targeting, rate of reduction of the DOI area, that practically represents the groundwater potential, assumes significance. Both the parameters, *k* and ∝, are important. While *k* relates to the quantum of groundwater filled fractures at the base of the weathered zone, ∝ determines how fast or slowly these fracture zones and associated Hydrolins reduce with depth. A high value of *k* and low value of ∝, for example, indicates an area with very good groundwater potential.

**Figure 5:**
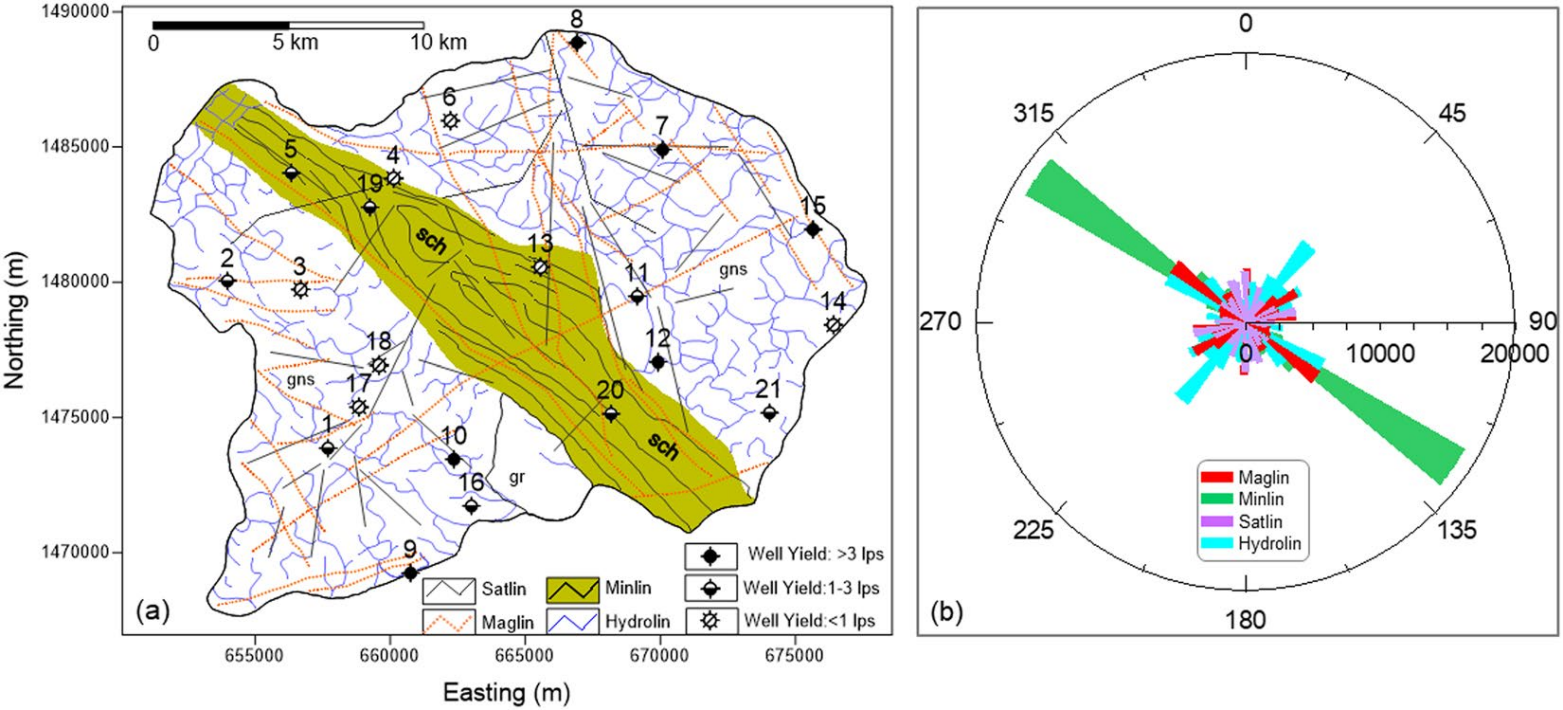
(**a**) Map showing distribution of different lineaments viz., Hydrolin, Satlin, Maglin and Minlin; (**b**) Directional characteristics of lineaments.

To understand any possible relationship between the Hydrolins and other lineaments, we have plotted all the lineaments together and studied their directional characteristics. Fig. 5a shows all the lineaments and wells. The Minlins are very distinct in the schist belt. The Satlins and Maglins are mostly linear and cut across geological formations displaying a regional nature. Hydrolins display curvilinear features due to inherent heterogeneities and crystal foliations in magma^69^, and anisotropic steering^70^.

The directions and lengths of various lineaments are plotted in the form of polar bar charts (Fig. 5b). Cumulative length of lineaments are calculated for various azimuths and then analyzed for their maximum, minimum and average lengths (Table 2) and matrix of their correlation coefficients is computed (Table 3).

**Table 2:** Statistical analysis of lineaments.

	Maglin	Minlin	Satlin	Hydrolin
Total Length (m)	135851	89283	117273	174083
Max. Length (m)	13320	37652	7763	14825
Min. Length (m)	1432	531	1420	285
Av length (m)	4382	6377	3554	4464
Numbers	31	27	35	206

**Table 3:** Correlation matrix of lineaments.

	Maglin	Minlin	Satlin	Hydrolin
Maglin	1	0.27	0.24	0.14
Minlin	0.27	1	0.01	0.32
Satlin	0.24	0.01	1	0.12
Hydrolin	0.14	0.32	0.12	1

The Minlins show strong directionality with maximum length among all. Whereas other lineaments have relatively more directional distribution. The Hydrolins show maximum directional distributions and have highest cumulative length. Their largest number (206) implies that they also have a good areal distribution (Table 2). The correlation matrix shows very poor correlation ranging from 0.32 to 0.01 for  correlation coefficients (CC). Out of these, Hydrolins show highest CC (i.e. 0.32) with Minlins and lowest (i.e. 0.12) with Satlins. Based on the directional characteristics and length, it is evident that Hydrolins are not influenced by any particular regional structural lineaments in the study area.

The Hydrolins describe the hydrogeological connectivity among groundwater bearing fracture zones. In contrast, the Satlins represent only the first few centimeters of the geomorphology, while the Maglins do not necessarily correlate with the high yielding wells. Therefore, we believe that Hydrolins qualify as more effective indicators of groundwater occurrence than Maglins or Satlins. Order of potentiality of these lineaments for groundwater prospects is shown in eq. (3) as follows:

3 $${\rm{Hydrolin}} > {\rm{Maglin}} > {\rm{Satlin}} > {\rm{Minlin}}$$

Yield data from a limited number of 21 bore wells in the watershed were analyzed and correlated with the AEM DOI. While additional wells are available in the watershed, we selected only those wells with authentic and verifiable records to ensure the veracity of results. Initially correlation coefficient is determined for all the well yields with corresponding AEM DOI and then successively by removing shallower DOI wells i.e. lowering the depth ranges viz., 20–250 m, 25–250 m, …, 200–250 m. The correlation coefficient for wells with all the DOI is found to be 0.48. It reduces to 0.43 and 0.41 for correlation over wells with range of DOI values 25–250 m and 30–250 m, respectively. The decreasing trend of correlation coefficient continues till the depth range 90–250 m. Thereafter correlation coefficient starts increasing to finally reach 0.9 for wells with DOI 170–250 m. A second order polynomial trend line fit shows the minimum at 80 m DOI, which can be considered as threshold depth beyond which deep DOI wells have better fracture connectivity and hence high yield potential. We term this depth as the ‘Threshold Groundwater Horizon’ (TGWH). In case of zones deeper than TGWH, chances of isolated fractures are significantly reduced and a clear positive correlation is achieved. The shallow (<80 m) zones comprise many hydrogeological complexities such as differential weathering and fracturing, reduction in the lateral connectivity, increased aquifer compartmentalization, etc. Some zones may have very good fracture connectivity, whereas others may be isolated fractured zones. Thus the wells at connected fractured pathways will experience increased yield with depth. Whereas isolated fractures may experience lowering of yield with depth.

**Figure 6:**
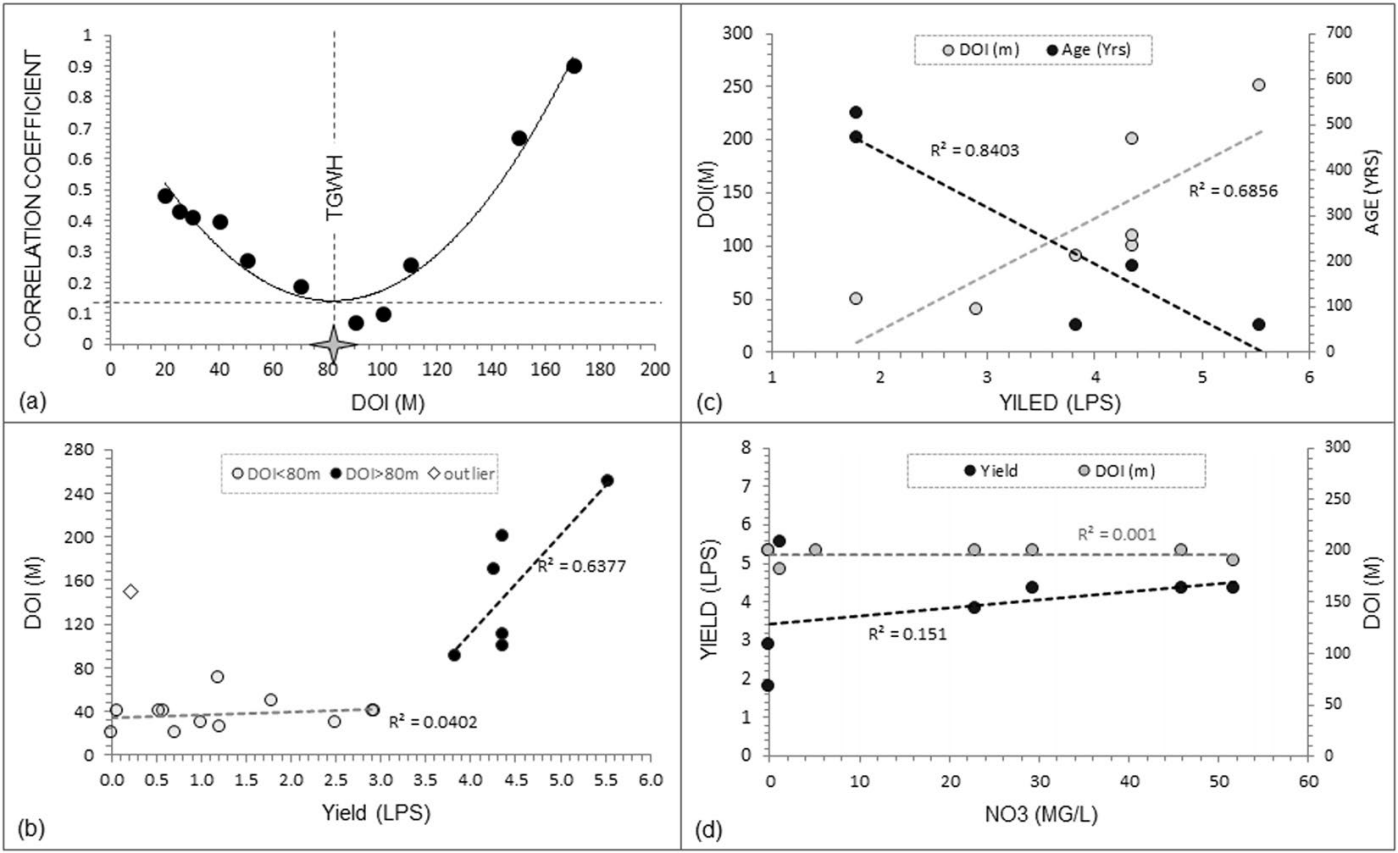
(**a**) Variation in correlation coefficient of well yield with DOI showing the Threshold Groundwater Horizon (TGWH) below which high yield wells can be located (**b**) Correlation of well yield with DOI, (**c**) Relationship of well yield with water age and DOI, (**d**) Relationship of water chemistry (NO3) with yield and DOI.

Figure 6b shows correlation of yield of wells with DOIs in two parts i.e. shallower wells above the TGWH and deeper wells. The correlation coefficient for shallower DOI-wells is found to be 0.04 that shows almost no correlation. However, the deeper DOI-wells reveal strong correlation (R^2^ = 0.64). The deeper zones beyond the TGWH are dominated by fractures with regional connectivity. While this result has useful implications, the trend beyond TGWH is based only on 7 data points. More data indeed would be useful to establish the exact nature of this trend.

Geochemical and isotope data are utilized to understand how Hydrolins control the hardrock hydrodynamics. Fig. 6c shows the regression relation of well-yield with groundwater age derived from C-14 dating and also with DOI. Well yield shows positive correlation (R^2^ = 0.68) with the DOI and negative correlation (R^2^ = 0.84) with the water age.

The bedrock fractures, in general, may have varying depth depending on local geological condition. In such conditions, groundwater occurrences are most likely expected to be more at deeper horizons under the influence of gravity. A deeper water saturated fracture will also increase the DOI. However, deeper well with strong interconnectivity and high transmissivity of fractures will facilitate movement of groundwater and hence will show lower age. In addition, C-14 dated age of water samples of the highest yielding well (i.e. KW12, Sasalu village) collected at the start of pumping and after 200 and 300 minute intervals have all shown modern age. This establishes the strong interconnectivity of fractures carrying fresh water recharged from the latest monsoon precipitation.

Although both natural and anthropogenic sources introduce NO_3_ contents into the groundwater, anthropogenic sources are recognized to be potential and most probable source causing increased nitrate content in groundwater. In such a case NO_3_ concentration ideally decreases with depth. However, because of strong lateral and vertical fracture interconnectivity and high transmissivity, no significant relationship of NO3 is seen with well yield and DOI (Fig. 6d). Even other major anions and cations do not have any noticeable correlations. Thus the above observation validates AEM derived Hydrolin as a potential groundwater pathway in crystalline hardrock.

The DOI provides the maximum depth limit of deep-seated conductive zones. This may mislead the interpreter to believe that there are continuous vertical fractures from the top to the maximum DOI. In fact, multiple sets of fractures could occur in a single well due to sets of horizontal to sub-horizontal and vertical to sub-vertical fractures. For example, in the existing 21 wells, the number of fracture sets varying from single (in KW3, 5, 11 & 13) to a maximum of seven (in KW12) have been encountered within a depth of 200 m (Table 1).

While DOI can be an effective indicator of the presence of water in deep fractures, it may be noted that a higher transmitter moment leads to a stronger S/N ratio and hence a deeper DOI. Thus a deep saturated fracture zone will produce a deeper DOI with an increase in the transmitter moment, indicating the presence of water at still deeper levels.

The results obtained by the SCI can be used in principle to create 3D images of subsurface electrical structures. However, we have used constrained inversions that assume the earth structures to be spatially coherent and the regularization concept results in an overall improvement of the resolution of geological structures that may not be well resolved by individual soundings^14^. A rigorous 3D inversion simultaneously considering the total survey area^71,72^ may improve the results. However, it would require extremely large computational resources in terms of memory, time and parallelization, particularly because fracture zones need very fine meshing. There is no software package currently available commercially to accomplish this objective.

## Conclusion

The present work emphasizes the importance of regional surveys in finding sustainable groundwater sources in hardrock terrains where most of the shallow wells located in the weathered zone have gone dry due to over exploitation and the challenge lies in locating sustainable groundwater sources in bedrock where limited secondary porosity is provided by sporadically distributed fracture zones.

The results demonstrate that the AEM surveys in combination with borehole data and geological information provide a potential tool to achieve this goal as they are capable of characterizing crystalline hardrocks and lineaments, mapping the spatial extent of fracture networks and associated hydrogeological pathways, termed as Hydrolins, that are derived from the AEM data. The DOI information efficiently demarcates the deep groundwater bearing fracture zones. Their yield potential is determined by the connectivity provided by the Hydrolins. The study also brings out a threshold depth, TGWH, below which the well-connected fracture zones are likely to provide high yielding wells. The AEM data also helps in studying the reduction in the groundwater bearing fracture zones with depth. The groundwater potential of a given watershed thus can be assessed by determining the availability of fracture zones below the TGWH. Lineaments are important in locating groundwater and the study reveals that Hydrolins provide the most significant clues followed by Maglins and Satlins. Additionally, a precise knowledge of Hydrolins can also be helpful in identifying suitable recharge zones. Knowledge of the fracture network in hardrock terrains is useful to understand regional hydrogeology and provide crucial inputs to simulate the groundwater flow system, and develop sustainable groundwater management plans. Since the study deals with the important problem of locating sustainable sources of groundwater in hardrocks, it is important that the present results supported by a limited number of groundwater data are further confirmed by expanding the analysis to larger groundwater data sets^73^.
